# Inactivation of Caliciviruses

**DOI:** 10.3390/ph6030358

**Published:** 2013-03-21

**Authors:** Raymond Nims, Mark Plavsic

**Affiliations:** 1 RMC Pharmaceutical Solutions, Inc.; 1851 Lefthand Circle, Suite A, Longmont, CO 80501, USA; 2 Corporate Product Biosafety, Genzyme, a Sanofi Company, 200 Crossing Boulevard, Framingham, MA 01701, USA; E-Mail: mark.plavsic@genzyme.com

**Keywords:** bovine calicivirus, canine calicivirus, chemical inactivation, feline calicivirus, human norovirus, murine norovirus, Norwalk virus, physical inactivation, San Miguel sea lion virus, vesicular exanthema of swine virus, vesivirus 2117

## Abstract

The Caliciviridae family of viruses contains clinically important human and animal pathogens, as well as vesivirus 2117, a known contaminant of biopharmaceutical manufacturing processes employing Chinese hamster cells. An extensive literature exists for inactivation of various animal caliciviruses, especially feline calicivirus and murine norovirus. The caliciviruses are susceptible to wet heat inactivation at temperatures in excess of 60 °C with contact times of 30 min or greater, to UV-C inactivation at fluence ≥30 mJ/cm^2^, to high pressure processing >200 MPa for >5 min at 4 °C, and to certain photodynamic inactivation approaches. The enteric caliciviruses (e.g.; noroviruses) display resistance to inactivation by low pH, while the non-enteric species (e.g.; feline calicivirus) are much more susceptible. The caliciviruses are inactivated by a variety of chemicals, including alcohols, oxidizing agents, aldehydes, and β-propiolactone. As with inactivation of viruses in general, inactivation of caliciviruses by the various approaches may be matrix-, temperature-, and/or contact time-dependent. The susceptibilities of the caliciviruses to the various physical and chemical inactivation approaches are generally similar to those displayed by other small, non-enveloped viruses, with the exception that the parvoviruses and circoviruses may require higher temperatures for inactivation, while these families appear to be more susceptible to UV-C inactivation than are the caliciviruses.

## 1. Introduction

The Caliciviridae are a family of non-enveloped, single-stranded RNA viruses with small particle size (27–40 nm). Like other small, non-enveloped viruses, the caliciviruses are able to survive under conditions that would be expected to inactivate other families of viruses (especially those that are enveloped). This relative resistance to inactivation is the subject of the present review.

The calicivirus family is comprised of four genera: *Vesivirus* (e.g.; feline calicivirus [FeCV], canine calicivirus [CaCV], bovine calicivirus [BoCV], vesicular exanthema of swine virus [VESV], San Miguel sea lion virus [SMSV], vesivirus 2117, and others); *Lagovirus* (e.g.; rabbit hemorrhagic disease virus and others); *Norovirus* (including the clinically important human noroviruses and murine norovirus [MNV]); and the clinically relevant *Sapoviruses*. There are also two proposed genera, the *Neboviruses* and the *Recoviruses* [1].

The caliciviruses and their respective inactivation susceptibilities (and resistances) have been studied intensively for years, due to the fact that this virus family represents the main cause of non-bacterial gastroenteritis in humans. An extensive literature exists for inactivation of various animal caliciviruses, especially feline calicivirus and murine norovirus. The latter have been studied as surrogates of the clinically more important human noroviruses since *in vitro* culture systems for the human noroviruses have not been commonly available. Systematic study of the inactivation of human noroviruses has therefore involved one or more of the three following approaches: (1) study of the responses obtained using surrogate viruses; (2) studies employing quantitative analysis of human norovirus genomic RNA using reverse transcriptase-polymerase chain reaction; and (3) clinical trials using human volunteers. There is ongoing debate as to the most appropriate approaches for studying the inactivation of the human noroviruses (more on this topic later in the review).

Interest in the caliciviruses moved beyond the food protection arena within the past decade as a result of another member of the *Vesivirus* genus, vesivirus 2117, which has been isolated from biologics manufacturing processes employing Chinese hamster cell substrates on several occasions, the first being reported in 2003 [2]. Additional occurrences of vesivirus 2117 contamination of biologics manufacturing were subsequently reported in 2009 [3]. The susceptibility of cells of different animal species to infection by this virus appears to be limited to the Chinese hamster, in which cells a progressive lytic infection ensues over a 14-day period [1]. The route of entry of this virus into biologics production processes has yet to be established with certainty, although the use of contaminated animal-derived materials, such as bovine sera, is considered to be the most likely source [2,4].

## 2. Literature Survey

As mentioned above, there is an extensive body of literature addressing the inactivation of caliciviruses, particularly those species that have served as surrogates for studying the inactivation of the clinically-important human noroviruses. There have been concerns expressed as to the appropriateness of utilizing one or more of these surrogate species in the study of human norovirus inactivation (until now it has been problematic to study the human noroviruses directly due to difficulties in culturing these viruses *in vitro*). A discussion of the various surrogate species and their relevance to human noroviruses, and their relative susceptibilities to various inactivation modalities may be found in Section 3.10 of this review. Within the biopharmaceutical industry it is accepted practice to use viral models of relevant contaminants in assessing purification strategies for viral inactivation and removal. In the case of the known contaminant, vesivirus 2117, any of the caliciviruses of genus *Vesivirus* would appear to represent acceptable model viruses for studying inactivation. The fairly extensive inactivation literature involving feline calicivirus (genus *Vesivirus*), should therefore be applicable to vesivirus 2117.

For the purpose of the present review, the various inactivation modalities that have been referred to as physical (*i.e.*, heat, UV, photodynamic/photocatalytic, ionizing radiation, and high pressure) have been discussed first, followed by a discussion of the chemical approaches. The latter have been arbitrarily subdivided to render the information more manageable. The various categories that are discussed include alcohols, oxidizing agents, other classes of chemical inactivants, and low pH.

Inactivation, by definition, refers to the elimination or reduction in infectivity of a virus. This is typically expressed as the log_10_ reduction in measured infectivity titer of a virus after exposure to inactivant. There have been reports of the measurement of genomic RNA as an endpoint for evaluating the inactivation of the human noroviruses, most typically in side-by-side studies with surrogate species for which both genomic material and infectivity have been measured. A great deal of effort has been expended in developing methods to derive meaningful inactivation information from evaluation of genomic RNA. This topic is discussed in more detail in Section 3.10 of this review.

Not all of the results from any given reference have been reproduced in the summary tables assembled within this review. This is particularly true of studies involving use of food products as coupons for studying viral inactivation, for high pressure processing methods applicable primarily to the food industry, and for certain proprietary disinfectants that are formulated with more than one class of inactivants. The reader is encouraged to consult the original references to access the entirety of the results contained therein. The authors also apologize, in advance, if we have inadvertently failed to mention one or more papers that rightfully should have been included in this review. We have made every effort to locate all papers that have addressed the inactivation of caliciviruses but doubt that we have been 100% successful in this respect.

For the present purpose, kinetic constants for thermal and chemical/disinfectant inactivation have not been typically available and therefore no attempt has been made to summarize such constants. On the other hand, inactivation through irradiation is more typically evaluated through dose-inactivation response studies and the results expressed as kinetic constants (*K*) or reduction factors such as *D*_37_ (fluence required for a 37% reduction in infectivity) and *D*_90_ or *D*_10_ (fluence required for a 1 log_10_ reduction in infectivity). We have chosen to express efficacy of inactivation in terms of the kinetic constant (*K*) expressed as log_10_ reduction in infectivity titer per unit fluence, in order to facilitate comparisons and to establish consensus values for the efficacy of the various irradiation inactivation modalities. Provided that log_10_ inactivation has been plotted against irradiation fluence, the *K* value may be obtained directly as the slope of the fit line. The *K* values obtained either directly in this manner or through conversion of *D* values may be interpreted as follows: a greater *K* value implies greater sensitivity of the virus to inactivation by the irradiation, while a lower *K* value implies greater resistance to inactivation.

For the most part, the mechanisms associated with the various physical and chemical viral inactivation approaches are considered to be out of scope for the present review. Where possible, references to literature discussing mechanism of inactivation have been provided. The one exception is for ionizing radiation. In this case, some discussion of the underlying mechanisms of inactivation was deemed to be required in order to interpret the results obtained by the various investigators.

## 3. Results and Discussion

### 3.1. Inactivation at Various Temperatures

The literature addressing the heat inactivation of caliciviruses [5,6,7,8,9,10,11,12,13,14,15,16,17,18,19,20,21,22,23,24,25] dates back to the early 1960s. The mechanisms underlying heat inactivation of single-stranded viruses have been discussed by Ginoza *et al.* [26], and Nuanualsuawan and Cliver [27] have addressed the mechanisms for the caliciviruses and picornaviruses in particular. A more recent discussion of mechanisms of heat inactivation of viruses of importance to food protection, including caliciviruses, can be found in Hirneisen *et al.* [28]. Heat inactivation of viruses may in some cases appear to be first-order with respect to contact time, but often graphs of log_10_ inactivation *vs.* time indicate departures from first-order kinetics [29]. It is rare that kinetic studies of the heat inactivation of caliciviruses have been complete enough to display this phenomenon, but good examples may be found in Buckow *et al.* [15] and Tuladhar *et al.* [23]. Due to the possibility for departure from first-order kinetics, the reporting of results solely in terms of decimal reduction values (*D*; time required to achieve a 1 log_10_ reduction in infectivity) for heat inactivation at a given temperature may be of marginal use in predicting extent of inactivation at times greater than that required for one log_10_ reduction. This problem is resolved when authors report both *D* values and the temperature *vs.* time plots from which the *D* values were obtained (e.g. [20]). It has been more typical for investigators to report inactivation results at one or a few temperatures rather than performing systematic kinetic studies. 

Selected results of studies of heat inactivation of caliciviruses are shown in Table 1. Heat inactivation data have been collected for FeCV, CaCV, MNV, and VESV. The data displayed for FeCV in Table 1 represent inactivation susceptibilities measured for four different strains (predominantly strain F9, but also strains KCD, 17FRV, and KS20). In addition, Ossiboff *et al.* [13] have evaluated and compared the temperature sensitivities of six different FeCV isolates (discussed in Section 3.10 of this paper).

**Figure 1:**
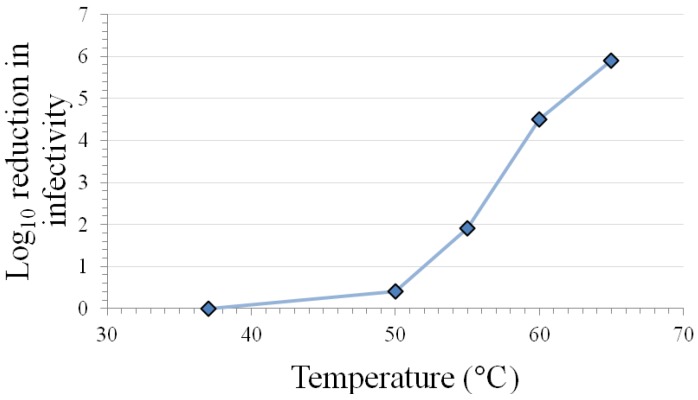
Inactivation of feline calicivirus strain F9 infectivity by 2 min heating at various temperatures in a Dulbecco’s phosphate-buffered saline matrix (modified from [17]).

The results in Table 1 indicate that the caliciviruses are inactivated at room temperature on surfaces and in solution after 7 days. Variable levels of inactivation occur at 50–56 °C in solution, with extensive inactivation requiring exposure times >30 min. Calicivirus species- and FeCV strain-differences in inactivation are observed at this temperature range (see Section 3.10). However, the temperature range 50–60 °C has been shown in plots of inactivation *vs.* temperature (see Figure 1) to include points within the steepest portion of the curve [17], so it may not be surprising that the highest variability in inactivation response occurs in this temperature range. More consistent and extensive inactivation is observed at temperatures in excess of 60 °C, regardless of calicivirus species or strain.

The caliciviruses appear to be resistant to the effects of freeze/thaw [9,25]. Five cycles of freezing and thawing resulted in only 0.44 log_10_ inactivation of CaCV and 0.34 log_10_ inactivation of FeCV [9].

Viral infectivity persistence on surfaces and/or in solutions at lower temperatures (4 °C, room temperature, 37 °C) is not the primary interest of this paper, although some data have been included in Table 1. Readers interested in additional information on this topic are referred to certain of the references [8,9,10,12,18,19,20,22,30,31]. 

**Table 1:** Inactivation of caliciviruses at various temperatures.

Inactivation Approach	Coupon material / Test matrix	Log_10_ Reduction in Infectivity Titer				Ref.
		FeCV	CaCV	MNV	VESV	
**Coupon Studies**						
25 °C for 7 days	Stainless steel	>5 *	−	~5	−	[12]
25 °C for 9 days	Stainless steel	−	−	~2.2	−	[19]
25 °C for 30 days	Stainless steel	−	−	6.2	−	[19]
**Solution Studies**						
25 °C for 7 days	Virus suspension	~3.5 *	−	>1	−	[12]
25 °C for 21 days	Tap water	≥6 *	−	−	−	[10]
37 °C for 7 days	Tap water	≥6 *	−	−	−	[10]
50 °C for 30 min	water	~3.5 ^†^	−	−	−	[6]
50 °C for 60 min	water	−	−	−	2–3	[5]
55 °C for 3 min	Virus stock	0.5 *	−	0.8	−	[24]
56 °C for 3 min	Virus stock	0 *	−	−	−	[8]
56 °C for 8 min	3-4 µg/mL protein virus stock	3 *	3	−	−	[9]
56 °C for 30 min	Virus stock	≥7 *	−	−	−	[13]
56 °C for 60 min	Virus stock	≥7.5 *	−	−	−	[8]
59 °C for 7 min	Virus + 10% FBS	4 ^‡^	−	−	−	[11]
60 °C for 5 min	Culture medium or water	~5 ^§^	−	−	−	[15]
60 °C for 10 min	Virus in PBS	−	−	~3.6	−	[22]
60 °C for 30 min	Virus in PBS	~2.1 *	−	2.2	−	[20]
62 °C for 30 min	Virus stock	≥7 *	−	−	−	[13]
63 °C for 10 min	Virus in water	-	−	3.3	−	[16]
65 °C for 2 min	Virus stock	>6.7 *	−	>6.7	−	[24]
**Inactivation Approach**	**Coupon material / Test matrix**	**Log_10_ Reduction in Infectivity Titer**				**Ref.**
		**FeCV**		**MNV**	**VESV**	
**Solution Studies**						
70 °C for 1.5 min	Culture medium or water	6 ^§^	−	−	−	[15]
70 °C for 2.5 min	Virus in PBS	−	−	~4.2	−	[22]
70 °C for 3 min	Virus stock	6.5 *	−	−	−	[8]
71 °C for 1 min	3-4 µg/mL protein virus stock	3 *	3	−	−	[9]
72 °C for 1 min	Virus stock	>6.7 *	−	>6.7	−	[24]
72 °C for 1 min	Virus in water	−	−	≥5	−	[16]
72 °C for 3 min	Virus in PBS	−	−	≥3.5	−	[18]
80 °C for 2.5 min	Culture medium or water	−	−	6.5	−	[14]

CaCV, canine calicivirus; FBS, fetal bovine serum; FeCV, feline calicivirus (*, strain F9; ^†^, strain 17FRV; ^‡^, strain KCD; ^§^, strain KS20); MNV, murine norovirus; PBS, phosphate-buffered saline; VESV, vesicular exanthema of swine virus.

### 3.2. Inactivation by UV Irradiation

Ultraviolet light has commonly been used in the disinfection of water [32], and more recently has been evaluated as a means of disinfecting food and food preparation surfaces [33,34] and as a barrier technology for mitigating risk of introducing a virus into a biomanufacturing process via contaminated process additives [35,36]. The mechanisms underlying UV inactivation of viruses in general have been discussed in [37], and additional references have addressed the UV inactivation specifically of caliciviruses and picornaviruses [27] and viruses of concern in food protection [28].

Ultraviolet radiation in the C range (UV-C; typically 254 nm) and the B range (UV-B; 280–320 nm) has been evaluated for efficacy in inactivating a variety of caliciviruses (Table 2). These studies [9,18,33,34,38,39,40,41,42,43,44] have included at least four different caliciviruses within inactivation matrices including both low protein and protein-containing solutions. The overall UV-C inactivation constant determined for caliciviruses from the data in Table 2 (*n* = 12) is 0.14 log_10_ reduction in titer per mJ/cm^2^ fluence. To put this value into perspective, a 4-log_10_ reduction in titer would be expected following exposure of a calicivirus to a fluence of 29 mJ/cm^2^.

Kowalski *et al.* [37] have used a prediction model based on the frequencies within the viral genomes of dimerizable base doublets (TT, TC, and CC) and triplets to estimate the *D*_90_ for UV-C inactivation of Norwalk virus to be 56 J/m^2^. This corresponds to a *K* of 0.18 log_10_ reduction in titer per mJ/cm^2^ (*K* = 1/*D*_90_; 10 J/m^2^ = 1 mJ/cm^2^). Lytle and Sagripanti [45] used a prediction model based on genome size to predict a *D*_37_ value for the calicivirus family of 36-40 J/m^2^. This corresponds to a *K* of 0.10 to 0.11 log_10_ reduction in titer per mJ/cm^2^ (*K* = 1/(*D*_37_ *2.43); 10 J/m^2^ = 1 mJ/cm^2^). Taken together, these empirical and predicted *K* values for calicivirus inactivation by UV-C indicate that a 4-log_10_ reduction in titer would require exposure of the viruses to 22–40 mJ/cm^2^ (220–400 J/m^2^). For comparison, a 4-log inactivation of parvoviruses would require exposure to 8-13 mJ/cm^2^ [37,45]; a 4-log_10_ inactivation of circoviruses would require 5–11 mJ/cm^2^ [45,46]; and a 4-log_10_ inactivation of polyomaviruses would require 240–370 mJ/cm^2^ [37,45,47].

The inactivation of caliciviruses by UV-C does not appear to be matrix-dependent under this (admittedly) limited set of conditions. The mean inactivation constants for low protein solutions (*K* = 0.14 log_10_ reduction in titer per mJ/cm^2^, *n* = 10) and higher protein solutions (*K* = 0.14 log_10_ reduction in titer per mJ/cm^2^, *n* = 2) are similar. The two values for the higher protein matrices fall within the 95% confidence interval (*K* = 0.11 to 0.17 log_10_ reduction in titer per mJ/cm^2^) for the ten low protein values. Although the data set in Table 2 does not include sufficient data points for MNV, BoCV, and CaCV to allow meaningful statistical analysis of inactivation susceptibility by species, it does appear that there is similarity in the mean *K* values obtained for the four species (range: 0.14 to 0.19 log_10_ reduction in titer per mJ/cm^2^).

Pulsed UV light in a Xenon Steripulse device has been used to inactivate MNV on food contact surfaces (stainless steel and polyvinyl chloride coupons) and in solution [34]. In the absence of added organic load, a 2-sec exposure at 10.5 cm (corresponding to ~50 mJ/cm^2^ fluence) from the broad spectrum (200–1,100 nm) UV light source inactivated 5 log_10_ of MNV on coupons and in solution, while the presence of organic load (5% fetal bovine serum) decreased the inactivation to ~3 log_10_.

**Table 2:** Inactivation constants for UV irradiation.

Irradiation Approach and Test Matrix	Inactivation Constant (*K*) and R^2^				Ref.
	FeCV ^a^	CaCV	MNV	BoCV	
**UV-C (254 nm) irradiation** (*K* = log_10_ reduction in titer per unit fluence [mJ/cm^2^])					
Water	–	–	–	*K* = 0.19 ^b^ R^2^ = 0.96 ^b^	[41]
Low protein virus stock, ambient temperature	*K* = 0.20 ^a^	*K* = 0.17 ^a^	–	–	[40]
	R^2^ = 0.96 ^a^	R^2^ = 0.93 ^a^			
Low protein virus stock, ambient temperature	*K* = 0.16 ^b^	–	*K* = 0.14 ^b^	–	[44]
	R^2^ = 0.99 ^b^		R^2^ = 1.00 ^b^		
Phosphate buffered saline, ambient temperature	–	–	*K* = 0.13 ^a^	–	[43]
			R^2^ = 0.96 ^a^		
Phosphate buffered saline, ambient temperature	*K* = 0.027 ^a^	–	–	–	[38]
	R^2^ = 0.75^a^				
Treated drinking water, ambient temperature	*K* = 0.12 ^b^	–	–	–	[39]
	R^2^ = 0.96 ^b^				
Buffered demand-free water, ambient temperature	*K* = 0.10 ^b^	–	–	–	[39]
	R^2^ = 0.92 ^b^				
Secondary effluent wastewater, ambient temperature	*K* = 0.21 ^a^	–	–	–	[42]
	R^2^ = 0.99 ^b^				
3–4 µg/mL Protein virus stock, ambient temperature	*K* = 0.13 ^a^	*K* = 0.15 ^a^	–	–	[40]
	R^2^ = 0.87 ^a^	R^2^ = 0.91 ^a^			
**UV-B irradiation** (*K* = log_10_ reduction in titer per unit fluence [mJ/cm^2^])					
3–4 µg/mL Protein virus stock, 4° C	*K* = 0.072 ^a^	*K* = 0.072 ^a^	–	–	[9]
	R^2^ = 0.98 ^a^	R^2^ = 0.96 ^a^			

BoCV, bovine calicivirus; CaCV, canine calicivirus; FeCV, feline calicivirus (all studies utilized strain F9); MNV, murine norovirus. ^a^ The values were calculated from the reported data. ^b^ Reported value.

A single publication [9] has reported data on the inactivation of caliciviruses (FeCV and CaCV) by ultraviolet radiation in the B range (280 to 320 nm). In an inactivation matrix of 3–4 µg/mL protein at 4 °C temperature, inactivation of both caliciviruses was observed with a *K* of 0.072 log_10_ reduction in titer per mJ/cm^2^ (Table 2). This indicates that a UV-B fluence of ~56 mJ/cm^2^ would be required to achieve a 4-log_10_ inactivation of these caliciviruses.

### 3.3. Photodynamic and Photocatalytic Inactivation

Photodynamic inactivation involves the addition of a photosensitizing (photoactive) agent such as methylene blue dye to solutions to be disinfected and the subsequent irradiation of the solutions with visible or ultraviolet light. This irradiation leads to the generation of singlet oxygen and oxygen radicals. Photodynamic inactivation has been investigated for a variety of potential bloodborne pathogens, primarily due to the interest in use of this inactivation approach for safeguarding the blood supply and achieving pathogen reduction in blood-derived products [48]. To our knowledge, the only report of the efficacy of a photodynamic approach for a calicivirus is the study of Mohr *et al.* [49]. A 5-minute illumination with visible light (45,000 Lux) following treatment of virus-spiked human plasma with 1 µM methylene blue dye resulted in >3.9 log_10_ inactivation of a calicivirus (species not specified in the paper).

Photocatalytic (or photoelectrochemical) inactivation has the advantage that it can be employed for disinfection of airborne viruses as well as viruses in solution. Photocatalytic inactivation involves the exposure of air streams or solutions to platinum-doped titanium dioxide (TiO_2_) in the form of particles or films. Upon irradiation of the photocatalyst with visible or UV light, complex photooxidants are generated which inactivate microorganisms through membrane damage and/or oxidative attack on internal macromolecules. Reports of the photocatalytic inactivation of caliciviruses have appeared in the past decade [43,50,51]. A combination of TiO_2_ particles and 254 nm UV light was found by Lee *et al.* [43] to effectively inactivate MNV (Table 3); however these authors did not find that TiO_2_ plus UV was more effective than UV treatment alone. Kato *et al.* [50] evaluated the efficacy of a TiO_2_ film plus UV or UV alone for inactivating human norovirus (NoV) in secondary wastewater effluent using reverse transcriptase-polymerase chain reaction to measure viral genomic RNA. These authors found that a combination of TiO_2_ and UV reduced levels of NoV genomic material by ~1 log_10_ (Table 3), while UV alone caused no reduction in levels of genomic material [50]. While the authors proposed that the combination of TiO_2_ and UV caused the decomposition of viral particles (and genomic RNA), this result was not obtained by Lee *et al.* [43]. In the latter study, neither TiO_2_ plus UV nor UV alone caused a reduction in genomic RNA levels. Inactivation of FeCV by visible light in the presence of a TiO_2_ film was investigated by Sang *et al.* [51]. The ~2 log_10_ reduction in titer observed required both TiO_2_ and visible light, and this inactivation was reduced to 0.9 log_10_ in the presence of added bovine serum albumin (BSA) (1 mg/mL).

**Table 3:** Photodynamic and photocatalytic inactivation of caliciviruses.

Irradiation approach and test matrix	Log_10_ reduction in titer			Ref.
	FeCV	NoV	MNV	
**Photodynamic Inactivation**				
1 µM methylene blue; human plasma, 5 min illumination	>3.9	–	–	[49]
**Photocatalytic Inactivation**				
TiO_2_ film plus visible light; virus stock, 24 hr at 30 °C	2.0 *	–	–	[51]
TiO_2_ film plus visible light, virus stock + 1 mg/mL BSA, 24 hr at 30 °C	0.9 *	–	–	[51]
TiO_2_ 10 mg/L plus UV 254 nm; virus stock, 3.8 min at ambient temperature	–	–	3.6	[43]
TiO_2_ film plus UV 254 nm; secondary effluent at ambient temperature	–	~1.0 ^a^	–	[50]

BSA, bovine serum albumin; FeCV, feline calicivirus (*, strain F4); MNV, murine norovirus; NoV, human norovirus. ^a^ Genomic material was assayed using reverse transcriptase-polymerase chain reaction; the value is likely therefore to be an underestimate of the reduction in infectivity.

### 3.4. Inactivation by Ionizing Radiation

The inactivation of viruses by ionizing radiation is virus-, dose-, matrix (scavenger)-, oxygen-, and temperature-dependent. Ionizing radiation has both direct effects (effects mediated by the gamma- or X-radiation itself) and indirect effects (effects mediated by oxygen radicals generated through radiolysis of water) [52]. The direct effects predominate under conditions where the temperature is very low (freezing and below), oxygen levels are relatively low, and there are relatively high amounts of scavengers of oxygen radicals (such as protein). On the other hand, irradiation at ambient temperature in the presence of oxygen and low levels of organic matter favor the indirect effects.

The literature on inactivation of caliciviruses by ionizing radiation (including gamma radiation and electron beam) is not extensive [40,53,54,55,56] (Table 4). The gamma radiation inactivation conditions evaluated have included low protein- and protein-containing matrices. These studies were performed at ambient temperature under conditions favoring the indirect effects of the ionizing radiation (*i.e.*; those resulting from radiolysis of water to form various oxygen radicals). The reported rate constants for inactivation are not consistent between investigators and there are not enough data points to make meaningful conclusions regarding efficacy, by species or by matrix, of inactivation by gamma irradiation.

There have been no studies published to date on the efficacy of gamma irradiation for inactivation of caliciviruses in frozen animal serum. The data shown in Table 4 that are considered to reflect primarily the indirect effects of gamma irradiation would not be considered representative of the efficacy of gamma irradiation in a frozen, highly scavenged matrix such as bovine serum, where the direct effects of gamma irradiation would be expected to predominate. On the basis of results obtained for other small, non-enveloped viruses [57], one might predict that the susceptibility of inactivation of caliciviruses (such as vesivirus 2117) in frozen bovine serum would be similar to that observed for picornaviruses (another family of non-enveloped single-stranded RNA viruses with similar particle sizes). If indeed this was the case, a *K* value of around 0.2 log_10_ reduction in titer per kGy fluence would be expected for inactivation of caliciviruses in frozen serum. This *K* value estimate corresponds to ~4 log_10_ inactivation at 20–21 kGy fluence. Unpublished results indicate that this level of calicivirus (FeCV) inactivation in frozen serum could be achieved at a gamma irradiation dose of 25-50 kGy.

There have been a few studies completed using electron beam for inactivation of caliciviruses in solution [54], in oysters and oyster homogenates [56], and on food surfaces (essentially analogous to a coupon study) [54,55]. The inactivation rates have ranged from 0.31 to 0.53 log_10_ reduction in titer per kGy in solution studies, and from 0.17 to 0.34 log_10_ reduction in titer per kGy in coupon studies (Table 4). This corresponds to a fluence of 7.5–13 kGy being required for a 4-log_10_ inactivation of a calicivirus in solution, and a fluence of 12–24 kGy being required for a 4-log_10_ inactivation of a calicivirus on a food surface.

**Table 4:** Inactivation constants for ionizing radiation.

Irradiation approach and test matrix	Inactivation constant (*K*) and R^2^			Ref.
	FeCV	CaCV	MNV	
**Gamma irradiation** (*K* = log_10_ reduction in titer per unit fluence [kGy])				
DMEM at ambient temperature	–	–	*K* = 0.33 ^a^	[53]
			R^2^ = 0.97 ^a^	
Low protein virus stock at ambient temperature	*K* = 5.9 ^a^	*K* = 7.4 ^a^	–	[40]
	R^2^ = 0.96 ^a^	R^2^ = 0.86 ^a^		
3–4 µg/mL Protein virus stock at ambient temperature	ND^c^	*K* = 7.0 ^a^	–	[40]
		R^2^ = 0.85 ^a^		
**Electron beam irradiation** (*K* = log_10_ reduction in titer per unit fluence [kGy])				
PBS at ambient temperature	–	–	*K* = 0.53 ^a^	[54]
			R^2^ = 0.99 ^a^	
DMEM at ambient temperature	–	–	*K* = 0.31 ^a^	[54]
			R^2^ = 0.98 ^a^	
Oyster homogenate	–	–	*K* = 0.20 ^a^	[56]
Whole oysters	–	–	*K* = 0.22 ^a^	[56]
Coupon (cabbage) at ambient temperature	–	–	*K* = 0.22 ^a^	[54]
			R^2^ = 0.98 ^a^	
Coupon (strawberry) at ambient temperature	–	–	*K* = 0.18 ^a^	[54]
			R^2^ = 0.93 ^a^	
Coupon (lettuce surface) at ambient temperature	*K* = 0.34 ^a^	–	–	[55]
	R^2^ = 0.98 ^b^			

CaCV, canine calicivirus; DMEM, Dulbecco’s modified Eagles Medium; FeCV, feline calicivirus (strain F9); MNV, murine norovirus; PBS, phosphate-buffered saline. ^a^ The values were calculated from the reported data. ^b^ Reported value . ^c^ ND, not able to be determined; dose-responsive inactivation was not observed in this study.

### 3.5. High Pressure Inactivation

Inactivation of viruses using high pressure represents a relatively recent addition to the list of physical inactivation modalities. The viral inactivation mechanisms underlying this approach have been discussed in references [28,58,59,60,61,62]. The approach has been evaluated primarily for use in food protection, with several studies reporting the efficacy of the approach for caliciviruses in particular (Table 5) [11,15,59,60,61,62,63,64]. More recently, high pressure has been employed in the biopharmaceutical industry as a means of dissociating protein aggregates and fostering protein refolding. While this has most often been utilized in microbial fermentation applications (e.g., [65]), it has also been used in mammalian cell culture processes [66]. Relevant factors that determine inactivation efficacy of high pressure processing are pressure, temperature, and time, as well as matrix water activity and pH [11,15,59,60,62].

**Figure 2:**
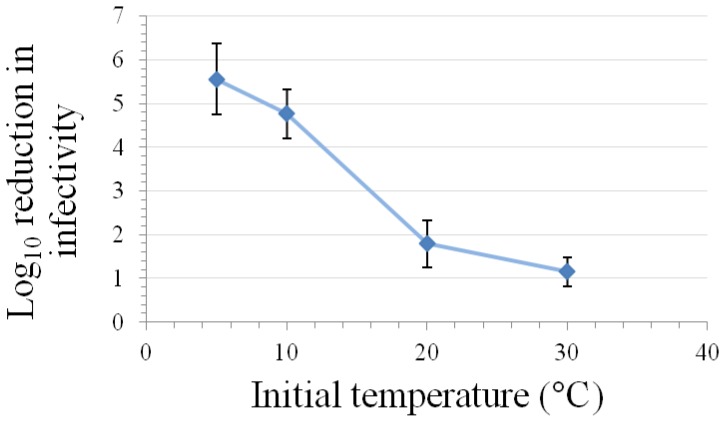
Effect of temperature on inactivation of MNV by a five-min high-pressure treatment at 350 MPa in a matrix of DMEM with 10% fetal bovine serum (mean ± SE, n = 9, modified from [60]).

Kinetic studies performed at room temperature and either 200–375 MPa pressure have demonstrated that the relationship between inactivation of caliciviruses and hold time at pressure is best modeled using a log logistic equation [11,60]. Buckow *et al.* [15] have used *n*th-order equations to model the time-dependence associated with the inactivation of FeCV at various temperatures and pressures. At both 200 and 250 MPa pressure, the greatest levels of inactivation are observed at temperatures of 0 °C or lower or at 50 °C or above, with the least inactivation being observed at room temperature [11]. This temperature dependency with regard to the time kinetics and/or extent of inactivation of FeCV or MNV at different pressures has also been observed by Buckow *et al.* [15], Lou *et al.* [62], and Kingsley *et al.* [60] (Figure 2). 

While fairly short exposure times at pressures in excess of 300 MPa readily inactivated FeCV and MNV in these studies, the results of a recent human volunteer study [64] suggest that higher pressures (600 MPa) and exposure times (5 min at 6 °C) may be required to completely inactivate human noroviruses.

**Table 5:** Inactivation of caliciviruses by high-pressure treatment.

Pressure	Temperature and Time	Test matrix	Log_10_ reduction in infectivity titer		
			FeCV	MNV	Ref.
200 MPa	−10 °C, 4 min	Virus stock in DMEM + 10% FBS	5.0 ^‡^	−	[11]
	0 °C, 4 min	Virus stock in DMEM + 10% FBS	4.4 ^‡^	−	[11]
	4 °C, 2 min	Virus + DMEM	−	~2.5	[62]
	4 °C, 5 min	Virus stock in DMEM + 10% FBS	4.7 ^‡^	−	[11]
	5 °C, 6 min	Virus + DMEM	3 ^§^	−	[15]
250 MPa	−10 °C, 4 min	Virus stock in DMEM + 10% FBS	4.6 ^‡^	-	[11]
	0 °C, 4 min	Virus stock in DMEM + 10% FBS	4.8 ^‡^	-	[11]
	4 °C, 2 min	Virus + DMEM	−	~5	[62]
	5 °C, 6 min	Virus + DMEM	~6 ^§^	-	[15]
300 MPa	4 °C, 2 min	Virus + DMEM	−	~5.4	[62]
	10 °C, 3 min	Virus + DMEM	~5 ^§^	−	[15]
	Ambient temp.; 3 min	Virus + cell culture medium	3.6 ^Φ^	−	[63]
350 MPa	5 °C, 5 min	Virus stock in DMEM + 10% FBS	−	5.6	[60]

DMEM, Dulbecco’s modified Eagles Medium; FBS, fetal bovine serum; FeCV, feline calicivirus (*, strain F9; ^‡^, strain KCD; ^§^, strain KS20; ^Φ^, strain 86/68); MNV, murine norovirus.

### 3.6. Inactivation by Alcohols

Alcohols, especially ethanol, are commonly employed as disinfectants in healthcare settings and in laboratory and manufacturing facility surface disinfection. Inactivation by alcohols of caliciviruses on coupons (including skin) and in solution has been addressed in numerous studies [8,9,31,67,68,69,70,71,72,73,74,75,76,77,78]. Selected calicivirus inactivation results determined in coupon studies (virus dried onto surfaces) and in solution studies have been displayed in Table 6.

The optimal concentration of alcohols (including ethanol, 1-propanol, and 2-propanol) for inactivation of FeCV appeared in the study of Gehrke *et al.* [67] to be 50%–70%, both when assessed in suspension and in coupon surface (skin) studies. In this study 1-propanol had the greatest efficacy of the three alcohols evaluated. Malik *et al.* [68] conducted coupon studies using stainless steel surfaces to evaluate the relative effectiveness of graded concentrations of ethanol or 2-propanol for inactivation of FeCV. In this study the optimal concentration for ethanol appeared to be in the range 70%–90%, and for 2-propanol 50%–70%, with contact times of 1–10 minutes [68]. The studies performed by Sattar *et al.* [78] on inactivation of FeCV and MNV on skin yielded similar results, with optimal inactivation resulting from 62%–75% ethanol. This result also is in general agreement with the results of Park *et al.* [74]. At this alcohol concentration range in this study and also in the study reported by Tung [77], MNV displayed greater susceptibility to inactivation by ethanol than FeCV (Table 6; see also Section 3.10). Belliot and coworkers [69] evaluated the efficacy of lower concentrations (10%–60%) of ethanol and 2-propanol for inactivation of MNV in solution studies. In either case, the 10% concentrations were ineffective, and at 30% only the 2-propanol with a 3-min contact time appeared to have some efficacy. A similar study was conducted by Magulski *et al.* [72] using MNV as the test organism, and lower concentrations (20%–60%) of ethanol, 1-propanol, and 2-propanol as inactivants. In this coupon (stainless steel) study, at the 30%–50% concentrations 1-propanol displayed the greatest efficacy of the three alcohols, and 2-propanol displayed weaker inactivation throughout the concentration range, relative to the other two alcohols [72]. Only the results for the 60% concentration have been shown in Table 6.

**Table 6:** Inactivation of caliciviruses by alcohols.

Inactivation Approach	Conditions	Coupon Material / Test Matrix	Log_10_ Reduction in Infectivity Titer			Ref.
			FeCV	CaCV	MNV	
**Coupon studies**						
Ethanol	62%; 30 s at ambient temp.	Skin	~2.1	−	~3.5	[78]
	70%; 30 s at ambient temp.	Skin	3.8	−	-	[67]
	75%; 30 s at ambient temp.	Skin	~2.3	−	~2.7	[78]
	75%; 30 s at ambient temp.	Skin	−	−	0.9	[71]
	80%; 30 s at ambient temp.	Skin		−	~1.7	[78]
	90%; 30 s at ambient temp.	Skin	2.8	−	−	[67]
	99.5%; 2 min at ambient temp.	Skin	1.3	−	−	[70]
	60%; 1 min at ambient temp.	Plastic	1.3	−	−	[75]
	60%; 5 min at ambient temp.	Stainless steel	−	−	~6.2	[72]
	70%; 10 min at ambient temp.	Stainless steel	1.3	−	−	[68]
	90%; 10 min at ambient temp.	Stainless steel	2.3	−	−	[68]
2-Propanol	60%; 5 min at ambient temp.	Stainless steel	−	−	~3.0	[72]
	70%; 10 min at ambient temp.	Stainless steel	2.3	−	−	[68]
	90%; 10 min at ambient temp.	Stainless steel	2.4	−	−	[68]
	60%; 1 min at ambient temp.	Plastic	<0.5	−	−	[75]
	70%; 30 s at ambient temp.	Skin	2.2	−	−	[67]
	90%; 30 s at ambient temp.	Skin	0.8	−	−	[67]
	91%; 2 min at ambient temp.	Skin	0.43	−	−	[70]
1-Propanol	60%; 5 min at ambient temp.	Stainless steel	−	−	~6.2	[72]
	70%; 30 s at ambient temp.	Skin	3.6	−	−	[67]
	90%; 30 s at ambient temp.	Skin	1.4	−	−	[67]
**Solution studies**						
Ethanol	30%; 3 min at ambient temp.	Virus stock + disinfectant	−	−	0.29	[69]
	50%; 30 s at ambient temp.	Virus stock + disinfectant	2.2	−	−	[67]
	50%; 5 min at ambient temp.	Virus stock + disinfectant	2.2	−	0.4	[74]
	50%; 20 min at 20 °C	Virus stock + disinfectant	<2	−	<2	[76]
	60%; 30 s at ambient temp.	Virus stock + disinfectant	−	−	>4	[69]
**Solution studies**						
Ethanol	70%; 30 s at ambient temp.	Virus stock + disinfectant	<0.5	−	~3.5	[77]
	70%; 30 s at ambient temp.	Virus stock + disinfectant	3.6	−	−	[67]
	70%; 2 min at ambient temp.	Virus stock + disinfectant	~1.2	~1.8	−	[9]
	70%; 5 min at ambient temp.	Virus stock + disinfectant	2.6	−	>3.6	[74]
	75%; 1 min at ambient temp.	Virus stock + disinfectant	1.3	−	−	[8]
	90%; 30 s at ambient temp.	Virus stock + disinfectant	<0.5	−	~3.6	[77]
	90%; 5 min at ambient temp.	Virus stock + disinfectant	0.3	−	>3.6	[74]
Sterillium^®^ gel	85% ethanol; 30 s at ambient temp.	Virus stock + disinfectant	−	−	>4	[69]
1-Propanol	30%; 20 min at 20 °C	Virus stock + disinfectant	>4	−	>4	[76]
	70%; 30 s at ambient temp.	Virus stock + disinfectant	≥4.1	−	-	[67]
2-Propanol	30%; 3 min at ambient temp.	Virus stock + disinfectant	−	−	1.6	[69]
	50%; 5 min at ambient temp.	Virus stock + disinfectant	0.8	−	1.0	[74]
	60%; 30 s at ambient temp.	Virus stock + disinfectant	−	−	3.9	[69]
	70%; 30 s at ambient temp.	Virus stock + disinfectant	2.4	−	−	[67]
	70%; 5 min at ambient temp.	Virus stock + disinfectant	0.2	−	>2.6	[74]
	90%; 5 min at ambient temp.	Virus stock + disinfectant	0.1	-	>2.6	[74]
Purell^®^ VF447	70%; 30 s at ambient temp.	Virus stock + disinfectant	≥4.8	-	≥3.7	[71]

CaCV, canine calicivirus; FeCV, feline calicivirus (all studies utilized strain F9); MNV, murine norovirus. Sterillium^®^ is a registered trademark of BODE Chemie GmbH & Co.; Purell^®^ is a registered trademark of GOJO Industries.

Added organic load (3% w/v bovine serum albumin + 3% w/v washed sheep erythrocytes [72] or human stool or artificial feces [77]) did not appear to have an impact on the inactivation of caliciviruses by alcohols, as demonstrated by the studies of Magulski *et al.* [72] and Tung [77].

### 3.7. Inactivation by Oxidizing Agents

Oxidizing agents are another useful category of viral inactivants that includes sodium hypochlorite (bleach) and other free chlorine- and iodine-generating agents, and active oxygen-generating agents such as hydrogen peroxide, paracetic acid, and ozone. The mechanisms underlying inactivation of caliciviruses and picornaviruses by chlorine are discussed in [27], and those underlying inactivation by chlorine, ozone, and chlorine dioxide of viruses of concern in food protection are reviewed in [28].

The literature that has included evaluations of inactivation efficacy of oxidizing agents for caliciviruses is extensive [8,9,18,31,42,69,70,72,73,75,77,79,80,81,82,83,84,85,86,87,88,89,90,91,92,93,94,95,96,97,98,99,100,101,102,103,104,105,106,107,108], due in part to the relative importance of the human noroviruses as human enteric pathogens and the need to identify efficacious agents for disinfection of food and food preparation surfaces. The calicivirus inactivation efficacy information for these agents determined in coupon studies (virus dried onto surfaces) and in solution studies is summarized in Table 7.

**Table 7:** Inactivation of caliciviruses by oxidizing agents.

**Inactivation Approach**	**Conditions**	**Coupon Material / Test Matrix**	**Log_10_ Reduction in Infectivity Titer**				**Ref.**
			FeCV	VESV	MNV	SMSV	
**Coupon Studies**							
Sodium hypochlorite	100 ppm; 10 min at 20 °C	Plastic	2.8 *	−	−	−	[85]
	1000 ppm chlorine; 1 min at ambient temp.	Plastic	>4.2 *	−	−	−	[75]
	1000 ppm; 10 min at 20 °C	Plastic	6.4 *	−	−	−	[85]
	3%; 10 min at ambient temp.	Stainless steel	−	−	≥4.8	−	[93]
	500 ppm free chlorine; 5 min at ambient temp.	Stainless steel	−	−	~2.3	−	[19]
	500 ppm free chlorine; 10 min at ambient temp.	Stainless steel	1.9 *	−	1.0	−	[100]
	800 ppm free chlorine; 10 min at ambient temp.	Stainless steel	1.1 *	−	−	−	[81]
	1000 ppm free chlorine; 5 min at ambient temp.	Stainless steel	−	−	≥6.2	−	[19]
	5000 ppm free chlorine; 2 min at ambient temp.	Stainless steel	3.2 *	−	1.5	−	[100]
	5000 ppm free chlorine; 10 min at ambient temp.	Stainless steel	3.4 *	−	−	−	[81]
Swan topical antiseptic	1% iodine; 30 s at ambient temp.	Skin	2.7 *	−	−	−	[70]
Hypochlorous acid	Fog; 5 min at ambient temp.	Ceramic tile	−	−	>3.5	−	[87]
**Coupon Studies**							
Hydrogen peroxide vapor	127 ppm; 1 hr at ambient temp.	Stainless steel	−	−	~5.2		[108]
	127 ppm; 1 hr at ambient temp.	Framing panels	−	−	~4.8		[108]
	Fog; 15 min at ambient temp.	Stainless steel	3.9 *	−	−	−	[101]
	Fog; 15 min at ambient temp.	Glass	≥5.2 *	−	−	−	[101]
	Fog; 15 min at ambient temp.	Vinyl flooring	≥5.2 *	−	−	−	[101]
	Fog; 15 min at ambient temp.	Plastic	≥5.2 *	−	−	−	[101]
Gaseous chlorine dioxide	8 ppm; 6 hr, ≥75% humidity, at 20 °C	Glass	>6 *	−	−	−	[91]
	8 ppm; 24 hr, 45%–55% humidity, at 20 °C	Glass	2.1 *	−	−	−	[91]
		Ceramic tile	≥5.2 *	−	−	−	[91]
Peracetic acid	1500 ppm; 5 min at ambient temp.	Stainless steel	−	−	~4.3	−	[72]
Ozone	20-25 ppm; 20 min at ambient temp.	Plastic	≥5.8	−	−	−	[86]
Axen30^®^	10 min at ambient temp.	Plastic	≥4.7 *	−	−	−	[104]
**Solution Studies**							
Betadine^®^	1% iodine; 30 s at ambient temp.	Virus stock + disinfectant	−	−	>4	−	[69]
Wescodyne®	0.02% iodine; 2 min at 25 °C	Virus stock + disinfectant	−	~4.5	−	≥6.3	[79]
Sani-Chick	0.8% iodine; 1 min at ambient temp.	Virus stock + disinfectant	≥5 *	−	−	−	[8]
Chlorine dioxide	0.2 ppm; 16 min at 20° C	Virus stock + disinfectant	~4 *	−	−	−	[90]
	0.29 ppm; 1 min at 5 °C	Demand free water, pH 7.2	−	−	≥3.5	−	[97]
	0.26 ppm; 30 s at 20 °C	Demand free water, pH 7.2	−	−	≥3.5	−	[97]
	0.4 ppm; 10 min at 20° C	Virus stock + disinfectant	~4 *	−	−	−	[90]
	0.8 ppm; 2.1 min at 20° C	Virus stock + disinfectant	~4 *	−	−	−	[90]
Dent-a-Gene	200 ppm; 10 min at ambient temp.	Virus stock + disinfectant	6.7	−	−	−	[82]
**Solution Studies**							
Sodium hypochlorite	0.1%; 2 min at 25 °C	Virus stock + disinfectant	−	≥6.3	−	≥6.3	[79]
	3%; 10 min at ambient temp.	Virus stock + disinfectant	≥6.7	−	−	−	[82]
	3%; 10 min at ambient temp.	Virus stock + disinfectant	≥6	−	−	−	[107]
	0.19 ppm; 2 min at 5 °C	Demand-free water atpH 7.2	−	−	≥2.5	−	[97]
	0.18 ppm; 1 min at 20 °C	Demand-free water at pH 7.2	−	−	≥2.5	−	[97]
	0.1 ppm free chlorine; 5 min at ambient temp.	Virus stock + disinfectant + 10 ng/mL protein	≥4.6 *	−	−	−	[89]
	0.1 ppm free chlorine; 5 min at ambient temp.	Virus stock + disinfectant + 120 ng/mL protein	3.5 *	−	−	−	[89]
	0.2 ppm free chlorine; 0.5 min at 5 °C	Buffered Water at pH 7 or 8	−	−	≥4	−	[94]
	0.2 ppm free chlorine; 0.25 min at 5 °C	Demand free tap water, pH 7	−	−	≥4	−	[94]
	0.5 ppm free chlorine; 15 s at 5 °C	Water at pH 6	≥4.3 *	−	−	−	[83]
	0.5 ppm free chlorine; 30 s at ambient	Treated water at pH 7.2	−	−	4.1	−	[95]
	2.5 ppm free chlorine; 10 min at ambient temp.	Seawater	−	−	~2.5	−	[102]
	8 ppm free chlorine; 5 min at ambient temp.	Primary effluent waste water	3.5 *	−	−	−	[42]
	75 ppm total chlorine; 30 s at ambient temp.	Virus stock + disinfectant	~1 *	−	~2.6	−	[77]
	250 ppm total chlorine; 30 s at ambient temp.	Virus stock + disinfectant	~2.5 *	−	~3.9	−	[77]
	300 ppm free chlorine; 10 min at ambient temp.	Virus stock + disinfectant	~1.5 *	−	-	−	[9]
	2600 ppm chlorine; 30 s at ambient temp.	Virus stock + disinfectant	−	−	>4	−	[69]
**Solution Studies**							
Sodium hypochlorite	3000 ppm free chlorine; 10 min at ambient temp.	Virus stock + disinfectant	>5 *	−	−	−	[9]
	5000 ppm chlorine; 1 min at ambient temp.	Virus stock + disinfectant	≥5 *	−	−	−	[8]
	5000 ppm free chlorine; 15 min at ambient temp.	Virus stock + disinfectant	>5 *	−	−	−	[88]
	5500 ppm free chlorine; 15 min at ambient temp.	Virus stock + disinfectant + 25% feces	4 *	−	−	−	[88]
Monochloramine	1ppm; 170 min at 5 °C	Buffered Water at pH 7 or 8	−	−	4	−	[92]
Trifectant^®^	1%; 10 min at ambient temp.	Virus stock + disinfectant	6.7	−	−	−	[82]
Potassium peroxy-monosulfate	1%; 2 hr at ambient temp.	PBS + disinfectant	≥7.1 *	−	≥6.6	−	[103]
Oxystrong FG	0.1%; 15 min at 20 °C	Virus stock + disinfectant + 40% FBS	≥3 *	−	−	−	[88]
Ozone	0.06 mg/L; 5 min at 5 °C	Water at pH 7	2.8 *	−	−	−	[84]
	1 ppm; 15 s at 5 °C	Water at pH 7	4.3 *	−	−	−	[84]
	1 ppm; 2 min at 20 °C	Demand free water at pH 7	−	−	~2	−	[96]
	1 ppm; 2 min at 20 °C	Demand free water at pH 5.6	−	−	~2.9	−	[96]
	6.3 ppm; 5 min at ambient temp.	Water	≥6.8 ^‡^	−	4.7	−	[99]

FBS, fetal bovine serum; FeCV, feline calicivirus (*, strain F9; ^‡^, strain KCD; ^§^, strain KS20); MNV, murine norovirus; SMSV, San Miguel sea lion virus; VESV, vesicular exanthema of swine virus. Axen30^®^ is a registered trademark of PURE Bioscience, Betadine^®^ is a registered trademark of Purdue Products, Dent-a-Gene is a trademark of Oxyfresh Worldwide, Oxystrong FG is a trademark of Ausimont GmbH, Sani-Chick is a trademark of Ruakura Pty Ltd, Swan is a trademark of Cumberland Swan, Trifectant^®^ is a registered trademark of Antec International Limited, Wescodyne^®^ is a registered trademark of Steris Corp.

The free chlorine-generating agents (sodium hypochlorite, Dent-a-Gene^®^, and hypochlorous acid) have been demonstrated to possess inactivation efficacy at relatively low free chlorine concentrations in solution studies. Urakami *et al.* [89] evaluated the sensitivity of FeCV to inactivation by free chlorine in the presence and absence of host cell debris and found that the log_10_ reduction in infectious titer achieved by 5 min exposure to free chlorine was highly dependent upon the chlorine demand of the matrix. In the more highly purified FeCV preparations, complete inactivation (≥3.2 log_10_) was achieved at 0.1 ppm free chlorine (5 min exposure at ambient temperature). This high degree of matrix-dependence as well as dependence on the aggregation state of the viruses for inactivation by free chlorine has also been discussed by Thurston-Enriquez *et al.* [83]. As shown in Table 7, numerous studies have evaluated the inactivation of caliciviruses by free chlorine levels from 0.5 ppm to 5,500 ppm in a variety of matrices, including primary effluent, seawater, and culture medium. In most cases, free chlorine levels in excess of 1,000 ppm have achieved complete inactivation of caliciviruses at relatively short contact times (~30 s). Longer contact times have been required to achieve inactivation of caliciviruses on coupon surfaces (Table 7).

Other oxidizing agents, including free iodine-generating agents (Betadine^®^, Wescodyne^®^, and Sani-Chick), gaseous chlorine dioxide, and active oxide-generating agents (hydrogen peroxide vapor, ozone, paracetic acid, Oxystrong FG, and Trifectant^®,^ and the proprietary stabilized silver compound, Axen30^®^) have demonstrated efficacy for inactivation of caliciviruses in solution studies and/or in coupon studies (Table 7). The mechanism underlying the inactivation of caliciviruses by the stabilized silver disinfectant [104] is likely similar to that of metallic copper. In the latter case, it appears that it is the solvated free cupper ions that mediate the inactivation of caliciviruses [105]. As with the other active oxygen-based disinfectants, the solvated copper ions generate reactive oxygen species and it is the latter that cause the observed oxidation of viral capsid proteins (105). The oxidizing agents that are in a form compatible with disinfection of facilities through fumigation (gaseous chlorine dioxide, ozone, hypochlorous acid, and vaporous hydrogen peroxide) appear to be equally effective for inactivating caliciviruses (Table 7). Of these, vaporous hydrogen peroxide and gaseous chlorine dioxide are the agents that typically have been used for facility disinfection following a viral contamination of a biopharmaceutical manufacturing facility with a small non-enveloped virus such as the calicivirus, vesivirus 2117 [109] or the parvovirus, mouse minute virus [110,111]. Gaseous chlorine dioxide must be used in conjunction with adequate humidity control, as the inactivation achieved is optimal at ≥75% relative humidity [91]. At lower relative humidity, much higher chlorine dioxide concentrations and contact times must be used to achieve similar levels of inactivation. The efficacy displayed by oxidizing agents for inactivating caliciviruses does not appear to be particularly species-dependent (Table 7; see also Table 10).

### 3.8. Inactivation by Other Classes of Disinfectants

There is extensive literature describing the inactivation of caliciviruses by other classes of disinfectants, including aldehydes [8,31,72,79,88,112,113,114], quaternary ammonium compounds [8,69,75,76,79,81,82,85,103,107,113], surfactants [8,69,73,112,113], sodium hydroxide [31,79], and other chemicals [103,115,116]. The calicivirus inactivation efficacy information for these agents determined in coupon studies (virus dried onto surfaces) and in solution studies is summarized in Table 8.

The effectiveness of inactivation of caliciviruses by the aldehydes, formaldehyde and glutaraldehyde, has been confirmed through several solution and coupon studies (Table 8). Blackwell [79] demonstrated in a solution study that the inactivation of VESV and SMSV by formaldehyde (1%) is very much temperature dependent, with greater inactivation occurring at 37 °C than at 25 °C and greater inactivation at 25 °C than at 4 °C. This temperature-dependence is not restricted to aldehydes, as it has also been observed for the quaternary ammonium compound, benzalkonium chloride [79]. The aldehydes have displayed similar inactivating efficacy for other families of small non-enveloped viruses, including the circoviruses [46], picornaviruses [27], and the polyomaviruses [47].

The efficacy of the quaternary ammonium-based disinfectants is compound- and condition-specific, with efficacy ranging from nil to complete inactivation of caliciviruses (Table 8). As mentioned above, Blackwell [79] found that a 20-min exposure of VESV and SMSV to 5% benzalkonium chloride in a solution study resulted in negligible inactivation at 25 °C, but complete inactivation at 37 °C. Kennedy *et al.* [107] investigated four proprietary mixtures of quaternary ammonium compounds for efficacy in inactivating feline calicivirus, and found that none of these completely inactivated the virus. The most efficacious was one composed of didecyl dimethyl ammonium chloride and N-alkyl dimethyl benzyl ammonium chloride that caused a 5-log_10_ reduction in titer (10 min contact time at ambient temperature) [107]. Whitehead and McCue [75] found that formulating a quaternary ammonium compound at higher pH (pH 12 *versus* pH 8) increased the efficacy of that compound for inactivating FeCV from <2.3 log_10_ to >3 log_10_. This result may, however, simply have reflected the inactivation of the calicivirus under strongly basic conditions, as a 2% solution of sodium hydroxide caused complete inactivation of VESV and SMSV [79].

A few proprietary surfactant disinfectants have been found to be highly effective for inactivating FeCV (Table 8), although it has been more typical to find that purely detergent-based disinfectants are relatively ineffective against caliciviruses [8,70]. This is what one would expect for a non-enveloped virus.

A few other chemical treatments that have displayed efficacy for inactivating caliciviruses include β-propiolactone (0.1%, 60 min at 22 °C [115]), and the proprietary alkylating agent INACTINE^®^ PEN 110 (0.1%, 3 hr at 22 °C [116]). These have displayed inactivation efficacy in very specific applications (viral inactivation in IgG [but not cryo-poor plasma], and red blood cell suspensions, respectively), and their utility for inactivation in other applications (matrices) has not been established. 

### 3.9. Low pH Inactivation

A low pH treatment is often included in downstream biologics manufacturing processes as a non-dedicated viral inactivation step. There is not an extensive literature dealing with low pH inactivation of caliciviruses, but that which could be found [6,9,12,21,31,75] is summarized in Table 9.

Many of the caliciviruses are enteric and, like the enteric picornaviruses, are adapted to survival in acidic environments. An exception to this is FeCV, which is a respiratory and not an enteric virus; and as might be expected, FeCV is more labile at low pH than the enteric caliciviruses (see Table 10 for additional comparative data for FeCV and MNV). The susceptibility of CaCV (typically considered to be an enteric virus) to low pH in the study of Duizer *et al.* [9] was perhaps surprising, and these data have been interpreted as evidence that CaCV is not a typical enteric virus. Indeed, true enteric canine caliciviruses more closely related genetically to human norovirus have been described [e.g.; 117] although these have yet to be evaluated for susceptibility to low pH or other inactivation modalities. The low pH inactivation results obtained for FeCV strain 17FRV displaying relatively high resistance to low pH treatment [6] are atypical for FeCV, and make one wonder if this virus was correctly identified.

**Table 8:** Inactivation of caliciviruses by other classes of disinfectants.

Inactivant and Conditions	Class of Disinfectant	Coupon Material/ Test Matrix	Log_10_ Reduction in Infectivity Titer				Ref.
			FeCV	MNV	VESV	SMSV	
**Coupon studies**							
Glutaraldehyde; 2500 ppm, 5 min at ambient temp.	Aldehyde	Stainless steel	−	~4.5	−	−	[72]
MicroQuat^®^; 1800 ppm, 10 min at ambient temp.	Quaternary ammonium	Stainless steel	2.3 *	−	−	−	[81]
Oasis^®^ 144; 1600 ppm, 10 min at ambient temp.	Quaternary ammonium	Stainless steel	2.0 *	−	−	−	[81]
UMQ; 3120 ppm, 10 min at ambient temp.	Quaternary ammonium	Stainless steel	3.4*	−	−	−	[81]
0.08%/0.02%, 10 min at ambient temp.	Quaternary ammonium	Stainless steel	−	~1.5	−	−	[93]
Formulation R-82; 0.39%, 10 min at ambient temp.	Quaternary ammonium	Plastic	6.5 *	−	−	−	[85]
1000 ppm; 1 min, pH 8, at ambient temp.	Quaternary ammonium	Plastic	<2.3 *	−	−	−	[75]
1000 ppm; 1 min, pH 12, at ambient temp.	Quaternary ammonium	Plastic	>3 *	−	−	−	[75]
**Solution studies**							
Formaldehyde; 0.7%, 30 min at 20° C	Aldehyde	Virus stock + disinfectant	≥2.3 *	−	−	−	[112]
Formaldehyde; 0.7%, 60 min at 20° C	Aldehyde	Virus stock + disinfectant	4.0 *	−	−	−	[113]
Formaldehyde; 0.7%, 30 min at 20 °C	Aldehyde	Virus stock + disinfectant	>4.0	−	−	−	[114]
Formaldehyde; 1%, 20 min at 25° C	Aldehyde	Virus stock + disinfectant	−	−	1.9	4	[79]
Glutaraldehyde; 0.5%, 1 min at ambient temp.	Aldehyde	Virus stock + disinfectant	≥5 *	−	−	−	[8]
Venno FF Super; glutaraldehyde, 0.1%, 15 min at 20 °C	Aldehyde	Virus stock + disinfectant + 40% FBS	≥3 *	−	−	−	[88]
**Coupon studies**							
Sodium hydroxide; 2%, 2 min at 4 °C	Base	Virus stock + disinfectant	−	−	≥6.3	≥6.3	[79]
Benzalkonium chloride; 5%, 20 min at 25 °C	Quaternary ammonium	Virus stock + disinfectant	−	−	<1	<1	[79]
Benzalkonium chloride; 5%, 20 min at 37 °C	Quaternary ammonium	Virus stock + disinfectant	−	−	≥6.3	≥6.3	[79]
Bacoban WB; 2%; 240 min at 20° C	Quaternary ammonium	Virus stock + disinfectant	≥4.0 *	−	−	−	[113]
A33^®^ Dry; 0.39%; 10 min at ambient temp.	Quaternary ammonium	Virus stock + disinfectant	1.0	−	−	−	[82]
Pine O Cleen; 0.15%; 1 min at ambient temp.	Quaternary ammonium	Virus stock + disinfectant	0 *	−	−	−	[8]
BARDAC^®^ 208M Blend; 1X, 30 s, pH 6.5, at ambient temp.	Quaternary ammonium	Virus stock + disinfectant	<0.5 *	<0.5	−	−	[77]
Asphène381^®^; 0.25%; 30 min at ambient temp.	Quaternary ammonium + surfactant	Virus stock + disinfectant	−	0.35	−	−	[69]
STERiZAR^®^ ; 80%; 5 min at 20° C	Surfactant	Virus stock + disinfectant	>4.0	−	−	−	[114]
Eradic8^®^ A2Z; 4%, 30 min at 20 °C	Surfactant	Virus stock + disinfectant	≥4.3 *	−	−	−	[112]
β-Propiolactone; 0.1%, 60 min at 22 °C	Lactone	IgG	5.2	−	−	−	[115]
β-Propiolactone; 0.1%, 300 min at 22 °C	Lactone	Cryo poor plasma	1.9	−	−	−	[115]
INACTINE^™^ PEN 110; 0.1%, 3 hr at 22 °C	Alkylating agent	RBC concentrates	−	−	≥7.5	−	[116]

FeCV, feline calicivirus (*, strain F9); MNV, murine norovirus; Asphène 381^®^ is a registered trademark of Laboratoire Rivadis, Bacoban is a trademark of Adexano^®^ GmbH, BARDAC^®^ 208M Blend and Formulation R-82 are registered trademarks of Lonza Inc.; Eradic8^®^ A2Z is a registered trademark of Amazon Bio-Guard Ltd.; Oasis^®^ 144, A33^®^ Dry and MicroQuat^®^ are registered trademarks of Ecolab, INACTINE™ PEN110 is a trademark of VI Technologies, Inc.; Pine O Cleen is a trademark of Reckitt and Benckiser, STERiZAR is a registered trademark of Creative Supply Solution Ltd.; UMQ is a trademark of Chemical Specialties Lab, Venno FF Super is a trademark of Menno Chemie Vertrieb.

**Table 9:** Low pH inactivation of caliciviruses.

pH	Acid, Temperature, Time	Coupon Material/ Test Matrix	Log_10_ reduction in infectivity titer			Ref.
			FeCV	MNV	CaCV	
**Coupon Studies**						
<1.0	0.38% Hydrochloric acid; 1 min at ambient temp.	Plastic	>5.0 *	−	−	[75]
1.6	0.38% Hydrochloric acid; 1 min at ambient temp.	Plastic	>3.2 *	−	−	[75]
2.5	0.25% Citric acid; 1 min, at ambient temp.	Plastic	>5.0 *	−	−	[75]
**Solution Studies**						
2	0.1 M Phosphoric acid; 15 min at ambient temp.	Virus in phosphate buffer	~4 ^†^	−	−	[6]
	0.1 M Citric acid; 30 min at 37 °C	Virus stock in DMEM	4.4 *	0.6	−	[12]
	Citric acid; 30 min at 37 °C	Virus stock in DMEM	>5 *	−	>5	[9]
	0.1 M Citric acid; 8.6 hr at 37 °C	Virus in PBS	-	1.0	−	[22]
2.5	0.1 M Phosphoric acid; 1 min at ambient temp.	Virus in phosphate buffer	>4 ^‡^	−	−	[6]
	0.1 M Phosphoric acid; 60 min at ambient temp.	Virus in phosphate buffer	~1.3 ^†^	−	−	[6]
3	0.1 M Citric acid; 15 min at ambient temp.	Virus in citrate buffer	~3.8 ^‡^	−	−	[6]
	Citric acid; 30 min at 37 °C	Virus stock in DMEM	~4.7 *	−	>5	[9]
	0.1 M Citric acid; 30 min at 37 °C	Virus stock in DMEM	3.7 *	0.6	−	[12]
4	0.1 M Citric acid; 30 min at 37 °C	Virus stock in DMEM	2.3 *	0.5	−	[12]
	0.1 M Citric acid, 22 hr at 37 °C	Virus in PBS	−	1.0	−	[22]

CaCV, canine calicivirus; DMEM, Dulbecco’s modified Eagles Medium; FeCV, feline calicivirus (*, strain F9; ^†^, strain 17FRV; ^‡^, strain C14); MNV, murine norovirus; PBS, phosphate-buffered saline.

*In vivo* infectivity studies using human volunteers [118] have demonstrated that Norwalk virus (human norovirus) retains infectivity after a 3-hr exposure to pH 2.7. In this respect, the enteric human noroviruses are more similar to the enteric surrogate MNV than to the non-enteric surrogate FeCV. Additional comments relating to the appropriateness of the various surrogates for studying inactivation of the human noroviruses can be found in the following Section 3.10.

### 3.10. Appropriateness of Calicivirus Surrogates for Studying Human Norovirus Inactivation

A variety of surrogate agents have been employed in the past to study the biology of the clinically important human noroviruses, for which *in vitro* infectivity systems have not been available. These surrogates have included animal viruses such as FeCV, MNV, San Miguel sea lion virus, bacteriophage (including MS2 and ΦX174), and norovirus virus-like particles [28]. For inactivation studies, which require an infectivity endpoint, the caliciviruses FeCV, CaCV, and more recently MNV have been the most commonly employed surrogates. Of these feline calicivirus has been used most often (see Table 1, Table 2, Table 3, Table 4, Table 5, Table 6, Table 7, Table 8, Table 9). Feline calicivirus, canine calicivirus, bovine calicivirus (BoCV), and San Miguel sea lion virus belong to genus *Vesivirus,* while MNV and the human noroviruses belong to genus *Norovirus*. In addition, FeCV is transmitted by the respiratory route, while MNV, BoCV, CaCV, and the human noroviruses are enteric and are naturally transmitted by the oral-fecal route. For these reasons, it has been suggested that MNV may be more appropriate as a surrogate for evaluating the biology of human noroviruses than FeCV [12,44,78,88,100,119]. In fact, it has been argued [120] that the various animal caliciviruses are not really appropriate surrogates for the human noroviruses on the basis of differences in inactivation susceptibility observed between different calicivirus species [77,120].

Side-by-side inactivation studies comparing FeCV and MNV have been conducted in an effort to identify potential differences that might exist in susceptibility to inactivation by various approaches [12,19,23,44,71,74,77,78,98,99,100,103]. The results of these studies have been summarized in Table 10. As might be expected, a difference in the resistance to low pH inactivation of these caliciviruses has been demonstrated. For instance, the enteric representative MNV was found to be much more resistant to inactivation at low pH (*i.e.*, pH 2–4) than the non-enteric FeCV [12]. Differences in susceptibility to 70%–75% ethanol between MNV and FeCV have also been identified, with MNV showing greater susceptibility in at least two studies [77,78]. Bleach (sodium hypochlorite) has been shown to be effective in inactivating caliciviruses. MNV displayed higher susceptibility to this agent than FeCV in side-by-side studies conducted by Tung [77], while FeCV displayed greater susceptibility in the side-by-side studies performed by Park and Sobsey [100]. There were methodological differences between these studies. For instance, the Park and Sobsey study evaluated sodium hypochlorite inactivation of viruses in 10% stool suspension dried onto stainless steel coupons, while the Tung study evaluated inactivation of viruses in solution without added organic load. The results described by Fraisse *et al.* [98] on reduction in titer of MNV and FeCV spiked onto lettuce leaves by various treatments have not been added to Table 10 since these reductions reflect both removal through washing and inactivation by peroxyacetic acid or sodium hypochlorite. Su and D’Souza [103] compared the inactivation responses of FeCV and MNV to the quaternary ammonium compound benzalkonium chloride and the oxidizing agent potassium peroxymonosulfate (Table 10). Each species displayed a similar response to the quaternary ammonium compound, while some differences in response to the oxidizing agent were observed at the lower doses (0.25 and 0.5 mg/mL). This difference was only observed in the presence of high titered virus stocks, not in the low titer virus stocks. The significance of this finding is not entirely clear. There were statistically significant differences (*p* < 0.05) noted in susceptibility to heat inactivation between FeCV and MNV at 56 °C but not at 63 °C and 72 °C [12]. Gibson and Schwab [20] reported that the *D* value for inactivation of FeCV at 50 °C (50.6 min) was significantly (*p* < 0.05) different than that for MNV (106 min), although the *D* values for the two viruses obtained at 60 °C (14.1 min *vs.* 13.7 min) were not significantly different. At this temperature range (~50–60 °C), differences in susceptibility were also noted for six different FeCV isolates, with log_10_ reductions ranging from ~3.0 to ~7.4 at 52 °C and ranging from ~5.3 to ~9.0 at 56.9 °C [13]. The differences in log_10_ reduction observed in side-by-side testing of MNV and FeCV inactivation by ozone (1 or 5 minutes exposure to 6.25 ppm at ambient temperature in a water matrix; Hirneisen *et al.* [99]; Table 10) were not statistically significant. In a side-by-side comparison [44], inactivation of FeCV and MNV by UV-C was found to display similar *K* values (0.16 *vs.* 0.14 log_10_ reduction in titer per mJ/cm^2^ fluence, respectively; Table 2). 

Side-by-side inactivation studies comparing feline calicivirus and canine calicivirus [9,40] and comparing San Miguel sea lion virus and vesicular xanthema of swine virus [79] have also been reported. The results of Duizer *et al.* [9] demonstrated that CaCV and FeCV display similar inactivation susceptibility to inactivation by heat (Table 1), UV-B (Table 2), and ethanol (Table 6). However, CaCV was more susceptible to inactivation by free chlorine from sodium hypochlorite than FeCV when tested in side-by-side studies. For instance, 300 ppm of free chlorine caused ~3.3 log_10_ inactivation of CaCV but only ~1.3 log_10_ inactivation of FeCV under similar conditions [9]. The study of de Roda Husman *et al.* [40] demonstrated that FeCV and CaCV are similarly sensitive to inactivation by UV-A and gamma irradiation (Table 2, Table 4). 

Blackwell [79] identified some differences in susceptibility of San Miguel sea lion virus and vesicular xanthema of swine virus to certain disinfectants. Most notable were the striking differences in susceptibility of these viruses to the iodine-containing disinfectant Wescodyne (0.02%; 2 min) and the substituted phenol-based disinfectants Amphyl (10%; 2 min) and One Stroke Environ (1%; 2 min). Vesicular xanthema of swine virus was very resistant to these disinfectants, especially at 4 °C, relative to San Miguel sea lion virus. On the other hand, the responses of the two viruses to formaldehyde (1%; 20 min), benzalkonium chloride (5%, 20 min), sodium hypochlorite (0.1%; 2 min) and phenol (5%; 2 min) were very similar [79].

Comparisons between MNV and human noroviruses [16,22,44,53,74,77,87,93,94,100,108] and FeCV and human noroviruses [9,24,44,74,77,86,88,100] have also been reported, although by necessity the evaluation of human norovirus inactivation in these studies has involved the evaluation of genomic RNA measured by RT-PCR, and not infectivity. Reductions in genomic content may not be reflective of inactivation, as genomic material from inactivated virus may still be detectable by RT-PCR. For this reason, it is difficult to derive meaningful conclusions as to differences or similarities in inactivation of these caliciviruses from such studies. Various methods of dissecting inactivation data from RT-PCR endpoint studies have been attempted, with various degrees of success [17,18,24,30,121,122,123]. Readers interested in the results of these comparisons between the inactivation of FeCV and MNV *versus* human norovirus as assessed with the RT-PCR endpoint, and in the methodological variations employed in extracting inactivation efficacy information from the RT-PCR endpoint are referred to Tung [77] and Rodriguez *et al.* [121].

**Table 10:** Side-by-side comparisons for inactivation of FeCV and MNV.

Inactivation approach and conditions	Log_10_ reduction in infectivity titer		Ref.
	FeCV	MNV	
Wet heat; 50 °C, 30 min	0.6 *	0.3	[20]
Wet heat; 55 °C, 3 min	0.5 *	0.8	[24]
Wet heat; 60 °C, 30 min	2.1 *	2.2	[20]
Wet heat; 65 °C, 2 min	>6.7 *	>6.7	[24]
Wet heat; 72 °C, 1 min	>6.7 *	>6.7	[24]
UV-C; in low protein virus stock, ambient temp. , 30 mJ/cm^2^	4.8 *	4.1	[44]
50% Ethanol; 5 min at ambient temp.	2.2 *	0.4	[74]
70% Ethanol; 30 s at ambient temp.	<0.5 *	~3.5	[77]
70% Ethanol; 5 min at ambient temp.	2.6 *	>3.6	[74]
Purell^®^ VF447; 70% ethanol, 30 s at ambient temp.	≥4.8 *	≥3.7	[71]
75% Ethanol; 20 s, on skin at ambient temp.	~0.8 *	~3	[78]
75% Ethanol; 30 s, on skin at ambient temp.	~2.4 *	~2.7	[78]
90% Ethanol; 30 s at ambient temp.	<0.5 *	~3.6	[77]
90% Ethanol; 5 min at ambient temp.	0.3 *	>3.6	[74]
Total chlorine; 75 ppm, 0.5 min at ambient temp.	~1 *	~2.6	[77]
Total chlorine; 250 ppm, 0.5 min at ambient temp.	~2.5 *	~3.9	[77]
Free chlorine; 500 ppm, 10 min at ambient temp.	1.9*	1.0	[100]
Free chlorine; 5000 ppm, 3 min at ambient temp.	4.5 *	2.8	[100]
Ozone; 6.3 ppm, 1 min at ambient temp.	2.7 ^‡^	3.9	[99]
Ozone; 6.3 ppm, 5 min at ambient temp.	≥6.8 ^‡^	4.7	[99]
Benzalkonium chloride; 0.1 mg/mL, 2 hr at ambient temp.	2.9 *	1.6	[103]
Benzalkonium chloride; 0.25 mg/mL, 2 hr at ambient temp.	3.1 *	2.3	[103]
Benzalkonium chloride; 0.5 mg/mL, 2 hr at ambient temp.	3.3 *	2.8	[103]
Potassium peroxymonosulfate; 2.5 mg/mL, 2 hr at ambient	≥7.1 *	0.9	[103]
Potassium peroxymonosulfate; 5 mg/mL, 2 hr at ambient	≥7.1 *	3.4	[103]
Potassium peroxymonosulfate; 10 mg/mL, 2 hr at ambient	≥7.1 *	≥6.6	[103]
pH 3; 30 min at 37 °C	3.7 *	0.6	[12]
pH 4; 30 min at 37 °C	2.3 *	0.5	[12]

FeCV, feline calicivirus (*, strain F9; ^‡^, strain KCD); MNV, murine norovirus.

## 4. Conclusions

As with inactivation of viruses in general, inactivation of the caliciviruses by the various physical and chemical approaches may be matrix-, temperature-, humidity-, and/or contact time-dependent. Substantial inactivation (*i.e.*, ≥4 log_10_) of caliciviruses in solution may be expected when using sufficient heat and contact time (>30 min at temperatures in excess of 60 °C), UV-C at fluence in excess of 40 mJ/cm^2^, UV-B fluence in excess of 60 mJ/cm^2^, high pressure (≥200 MPa for ≥5 min at 4 °C), formaldehyde (≥7,000 ppm for ≥30 min at ambient temperature) and free chlorine in excess of 2,500 ppm (≥30 s at ambient temperature). For disinfection of surfaces, electron beam irradiation (fluence > 20 kGy at ambient temperature), glutaraldehyde (≥2,500 ppm for ≥5 min at ambient temperature), and fogging with hypochlorous acid (≥5 min at ambient temperature), hydrogen peroxide vapor (≥15 min at ambient temperature), or ozone (20–25 ppm for ≥20 min at ambient temperature) should afford ≥ 4-log_10_ inactivation of caliciviruses.

Differences between calicivirus species are observed with respect to some, but not all of the inactivation modalities. The most striking differences are in susceptibility to inactivation by low pH and ethanol, while susceptibility to inactivation by physical means (UV-B, UV-C, gamma irradiation, and heat) appears to vary to a lesser extent among the various calicivirus species. Due to the observed species differences in susceptibility to inactivation to these agents, alcohols and quaternary ammonium-based disinfectants, and low pH may not afford adequate inactivation of all calicivirus species of concern.

Until infectivity endpoints are developed for human noroviruses, the enteric calicivirus MNV would appear to represent the most appropriate surrogate for studying inactivation of the human noroviruses. As observed with various FeCV strains and field isolates (Table 1, Table 2, Table 3, Table 4, Table 5, Table 6, Table 7, Table 8, Table 9, Table 10 and ref. [13], different human norovirus isolates may also turn out to display differing responses to inactivation modalities when additional clinical trials are performed using human volunteers, as has been advocated by Richards [120].

In summary, the susceptibilities of the caliciviruses to the various physical and chemical inactivation approaches are generally similar to those displayed by other small, non-enveloped viruses, with the exception that the parvoviruses and circoviruses may require higher temperatures for inactivation, while these families appear to be more susceptible to UV-C inactivation than are the caliciviruses.
